# Characterization and RNAi-mediated knockdown of Chitin Synthase A in the potato tuber moth, *Phthorimaea operculella*

**DOI:** 10.1038/s41598-017-09858-y

**Published:** 2017-08-25

**Authors:** Ahmed M. A. Mohammed, Mervat R. Diab, Mohamed Abdelsattar, Sayed M. S. khalil

**Affiliations:** 1 Agricultural Genetic Engineering Research Institute (AGERI), Agricultural Research Center, 9 Gamaa street, Giza, Egypt; 2 Plant Protection Department, College of Food and Agriculture Sciences, King Saud University, Riyadh, Saudi Arabia

## Abstract

Chitin is a major component of insect exoskeleton, tracheal system and gut where it is synthesized by chitin synthase (CHS) enzymes. In this paper, we report the isolation and RNAi of chitin synthase A (*PhoCHSA*) from the potato tuber moth *Phthorimaea operculella*. The full-length cDNA of *PhoCHSA* is 5,627 bp with 4,689 bp open reading frame coding for 1,563 amino acids. Structural analysis of conceptual amino acid translation showed three distinct regions found in all known insect CHS proteins; N-terminus region having 9 transmembrane helices, middle catalytic region containing several conserved domains identified in insect CHS enzymes, and C-terminus region containing seven transmembrane spans. Phylogenetic analysis showed that PhoCHSA protein clustered with CHSA enzymes identified from insects from different insect orders. RNAi targeting three different regions of the gene showed different efficacy against potato tuber moth larvae and dsRNA targeting the 5′ region has the highest efficacy. Results were verified by qRT-PCR which showed that dsRNA targeting the 5′ region caused the highest reduction in *PhoCHSA* mRNA level. Our results show the importance of selecting the RNAi target region and that chitin synthase A can be a suitable RNAi target for the potato tuber moth control.

## Introduction

Chitin is a polysaccharide chain of β-(1,4)-*N*-acetyl-D-glucosamine (GlcNAc) and is found in the cell walls of fungi and in the exoskeletons of arthropods including insects. It is a structural component of insect cuticle, in the exoskeleton, and the peritrophic matrix (PM) that lines the midgut lumen^1, 2^. Chitin is synthesized by chitin synthase (CHS) enzyme which transfers GlcNAc residue to the growing polysaccharide chain. Two CHS classes have been identified in insects; CHSA is responsible for biosynthesis of the chitin found in the cuticular exoskeleton and other tissues such as the foregut, hindgut and trachea, whereas chitin associated with the PM is synthesized by CHSB^3–6^.

Full-length cDNA of CHSA was first isolated from the Australian sheep blowfly, *Lucilia cuprina* ^3^, then followed by a number of CHS genes identified from different insects summarized by Merzendorfer^7^ and Muthukrishnan *et al*.^8^. Recently, more CHS genes were identified from other insects such as *Bactrocera dorsalis* ^9^, *Bombyx mori* ^10^ and *Toxoptera citricida* ^11^. Insect chitin synthases are large transmembrane proteins with theoretical molecular masses of about 170-180 kDa and consist of three structural domains; the N-terminal domain (domain A), the central catalytic region (domain B) and the C-terminal domain (domain C)^7^. Domain A exhibits 7–10 transmembrane helices and depending on its number, the N-terminus faces either the extracellular space or the cytoplasm^12^. Domain C includes seven transmembrane spans of which five are located close to the catalytic domain and are believed to play a role in chitin extrusion. Domain B is conserved among CHS proteins and is involved directly in synthase catalysis^6^. The catalytic domain shares some conserved motifs necessary for enzyme function such as the nucleotide binding, donor saccharide binding, acceptor saccharide binding and product binding sites^6^. Motifs such as “DXD”, “EDR”, “CATMWHXT” and “QRRRW” contribute to divalent cation binding, catalysis, and substrate binding, respectively. The growth and metamorphosis of insects depend strictly on structural changes in the tissues and organs that contain chitin such as the epidermis, trachea and PM of the midgut. Several enzymes included in the chitin synthesis, modification and degradation have been identified and their roles have been explained and reviewed by Zhu *et al*.^13^. Therefore, chitin metabolic pathway provides promising target for novel insecticides^14^.

RNA interference (RNAi) is a mechanism of post-transcriptional regulation of gene expression in higher eukaryotes^15^. It was first discovered in *C*. *elegans* by Fire *et al*.^16^. RNAi has been applied in a variety of insect orders including Coleoptera, Hemiptera, Diptera, Hymenoptera and Lepidoptera^17–20^. The successful application of RNAi in pest management depends mainly on two key factors; the selection of the target gene and the delivery method^21^. Target gene should be lethal to the pest upon silencing meanwhile should be safe to non-targets such as natural enemies and human being. Several target genes coding for functional and structural proteins were evaluated as targets for insect control and showed different levels of success summarized by Kola *et al*.^22^. The application of RNAi in most species is limited by the delivery method of dsRNA into the target cells. Direct injection of dsRNA into insect hemocoel is the common method of dsRNA delivery^23, 24^. Oral delivery methods such as droplet feeding, mixing with artificial diet and feeding on bacterially expressed dsRNA have been successfully used in a number of species^13, 20, 25^. In other species ingestion of dsRNA failed to induce RNAi^26^. Baum *et al*.^17^ and Mao *et al*.^27^ were the first to describe successful RNAi-mediated insect control through transgenic plants expressing dsRNA targeting insect genes. Joga *et al*.^28^ discussed the advantages and disadvantages of different methods used for dsRNA delivery to insect pests such as nanoparticles, root soaking, trunk injection, microorganisms and transplastomic plants. RNAi for CHS genes was achieved in a range of insects including sucking^29^, chewing^30^ and blood feeding insects^31^ through virus-infected plants, transplastomic plants and injection, respectively. CHS RNAi in those cases showed promising success in insect control.

*Ph. operculella* (potato tuber moth, PTM) is a serious insect pest of the potato crop in tropical, subtropical and Mediterranean regions. Main infestation occurs during storage period where healthy and infested tubers are mixed together. Hence, severe infestations result in yield and quality losses during storage^32, 33^. In Egypt, PTM has caused up to 100% losses to potato plants in fields as well as in storage^34^. In the present article, we cloned chitin synthase class A from PTM larvae. The effect of dsRNA on silencing CHSA was determined by injecting PTM larvae with three dsRNA molecules targeting three different regions of the gene. Although the three dsRNA molecules target the same transcript, mortality was variable according to the target region. Results were confirmed by qRT-PCR which showed that silencing effect was also variable according to the target region.

## Materials and Methods

### Insect Colony

PTM infected tubers were obtained from the colony maintained in the insect rearing facility at Agriculture Genetic Engineering Research Institute. The infected tubers were added to dry clean potato tubers in plastic trays covered with muslin mesh. The trays were supplied by cardboard stacks, providing places for pupation, and kept at 26 ± 2 °C. Cardboard stacks containing pupae were transferred into glass jars covered with filter papers serving as oviposition sites for emerged adults.

### cDNA cloning of Chitin synthase A

*Ph*. *operculella* larvae were dissected, guts were removed, and the rest of the larval bodies was used to isolate total RNA using Trizol^®^ reagent (Invitrogen, Carlsbad, CA, USA). First strand cDNA was prepared using the Superscript II cDNA synthesis kit (Invitrogen) according to the manufacturer’s instruction. Three degenerate primer sets (Table 1) were designed based on the conserved regions of *CHSA* nucleotide sequences deposited in the GenBank database from other insect species. Those primers were used to amplify the target *CHSA* regions in a standard PCR reaction using the first strand cDNA as a template. PCR amplified fragments were cloned into pGEM-T Easy vector (Promega, Madison, WI, USA) and transformed into DH10β chemically competent cells (Invitrogen). Following bacterial overnight growth and plasmid DNA purification, the cloned fragments were subjected to sequence analysis by the facility of Macrogen Korea (Seoul, Republic of Korea). New three pairs of gene-specific primers (Table 1) were designed based on the sequence of initial fragments and were used to obtain the intermediate regions of the message using cDNA as a template. Both 5′ and 3′ ends were synthesized using First Choice^®^ RLM-RACE kit (Ambion, Austin, TX, USA). All PCR products were treated as explained before. The resulted sequences were assembled together using ContigExpress utility of Vector NTI Advance 11 (Invitrogen) to obtain the full-length *PhoCHSA* cDNA sequence.

**Table 1:** Nucleotide sequences of primers used in cloning, RACE, dsRNA synthesis and real time PCR analysis.

Primer	Sequence 5′–3′
**Cloning primers**	
CHSA201FD	CGSCGSGADGVVGGHAG
CHSA311RD	CTGTCCTGCTTCGGHGGRAWYTCYCGG
CHSA2529FD	GGYCATTGGCTGCARAAGGC
CHSA3072RD	GCGCCACCARTAACTGYA
CHSA4209FD	GCYATGTTGTTCCATMGATTYGAAAC
CHSA4440RD	CTCTTCTTCTGYTTNGCYTTYTC
CHSA214FS	GAAGCGATAACTCCGACGACGA
CHSA2679RS	CCAGCTATCCTCACCTTGATCGTACTG
CHSA1551RS	CCAAACCCAAGCCATTTGACGCGA
CHSA1345FS	GCTACATTTTTGGAAAGTTCGC
CHSA3207FS	GCGCTCTCAAGCTCATTCTT
CHSA4388RS	GTCGTTGTCGTAGTCGTCATCCAG
**RACE primers**	
**5′-end**	
CHSA225RS	GGTGTGAGCTCGTCGTCGGA
CHSA216RS	TCGTCGTCGGAGTTATCGCT
**3′-end**	
CHSA4202 FS	GTTCACTGCTATGTTGTTCCATCG
CHSA4365FS	CTGGATGACGACTACGACAACGAC
**dsRNA primers**	
CHSB-F 2177	TAATACGACTCACTATAGGGCTCCATATGGAGGGAGACTT
CHSB-R 2663	TAATACGACTCACTATAGGGTTCGTCAGATCGCAAAGTGT
CHSA-F 314	TAATACGACTCACTATAGGGAGAAATCCCTCCGAAGCAGGACA
CHSA-R 834	TAATACGACTCACTATAGGGCCGCGAGCTTCGAGCCAAAAGA
CHSA-F 3458	TAATACGACTCACTATAGGG AGGATTCCTCCAAGGCGCGAAC
CHSA-R 3957	TAATACGACTCACTATAGGGCCCTGGAAATACGAGCCTGTTCCT
AMPF	TAATACGACTCACTATAGGGCCCAGTGCTGCAATGATACC
AMPR	TAATACGACTCACTATAGGGTTCTGACAACGATCGGAGGAC
**qRT-PCR primers**	
CHSRTF-361	GCCTGGAGTTCACAGTCAGA
CHSRTR-489	GCCGGTCTTTCTTAAGTTGC
ACTF-135	CGTSTTCCCSTCCATCGTVGG
ACTR-419	ACATGATCTGSGTCATCTT

### Sequences and phylogenetic analysis

The sequence of *PhoCHSA* cDNA was compared with other sequences deposited in GenBank using the “BLASTN” and “BLASTX” tools at the National Center for Biotechnology Information (NCBI) web site (http://blast.ncbi.nlm.nih.gov/Blast.cgi). Alignments of nucleotide sequences and deduced amino acid sequences from cDNA clones were done with the aid of the Clustal Omega software (http://www.ebi.ac.uk/Tools/msa/clustalo/). The molecular weight (MW) and isoelectric point (pI) were calculated computationally on ExPASy Proteomics website (http://web.expasy.org/cgi bin/compute_pi/pi_tool). The deduced amino acid sequence was scanned for motifs against the PROSITE database (http://prosite.expasy.org). Transmembrane helices and glycosylation sites were predicted using TMHMM (v2.0) (http://www.cbs.dtu.dk/services/TMHMM-2.0/) and NetNGLyc 1.0 software (http://www.cbs.dtu.dk/services/NetNGlyc/). Also the sequence was scanned using YinOYang program (http://www.cbs.dtu.dk/services/YinOYang/). The phylogenetic tree was constructed using the neighbor-joining method in MEGA version 7.0^35^.

### dsRNA synthesis

The dsRNA fragments were prepared by *in vitro* transcription using MEGAscript^®^ RNAi Kit (Ambion) according to manufacturer’s instructions. Three dsRNA fragments: 5′-dsRNA, Mid-dsRNA and 3′-dsRNA were prepared to target the 5′, middle and 3′ regions of the *PhoCHSA* transcript, respectively. The numbers supplied with each primer pair (Table 1) represent the region to be targeted by the dsRNA, where in all cases a region around 500 bp was selected. Ampicillin resistance gene specific dsRNA (dsAmpR) was prepared using AMPF and AMPR primer set (Table 1) to be used as a control. Each primer contains the T7 promoter sequence (TAATACGACTCACTATAGGG) at the 5′ end that is necessary for RNA synthesis. The dsRNA produced was eluted in ddH_2_O, quantified by spectrophotometer and stored at −20 °C till injection.

### Bioassay of dsRNA Fragments

The lethal effect of RNAi was evaluated by delivering three different amounts (50, 100 and 200 ng) of three independent dsRNA fragments (5′-dsRNA, Mid-dsRNA and 3′-dsRNA) into larval hemocoel by direct injection. On day 2, similar sized third instar larvae were collected from the infested tubers and were injected using Neuros Syringe model 1701RN controlled with a dispenser (Hamilton, Höchst, Germany). Larvae were kept on ice to reduce their movements before injection. A group of 45–50 third instar larvae were injected in each treatment and the injection was repeated four times for each replicate. Each replicate was injected in a separate day. Control larvae were injected with 200 ng of dsAmpR. Each replicate of injected larvae was placed on a clean sliced potato tuber and kept at the same rearing conditions with the main colony. Larvae died within the first 24 h post-injection were removed and not counted. The total mortality was recorded five days after injection. Statistical analysis of the mortality data was performed with Student’s t-test in the Excel program.

### Quantitative real time PCR (qRT-PCR)

#### The expression pattern of PhoCHSA

The expression levels of PhoCHSA were detected using real-time PCR in eight days during larval development between the last day of 2nd and 4th instars. The total RNA was isolated from epidermal tissues collected from 4–7 larvae per day and the expression levels of PhoCHSA were measured.

#### RNAi

*PhoCHSA* mRNA level was quantified by qRT-PCR in *Ph*. *operculella* larvae injected with one of the three *CHSA* specific dsRNAs. For each dsRNA fragment, a group of 50–60 of two-day-old 3rd instar larvae was injected with 200 ng dsRNA and was added to single clean sliced tuber. In addition, another group was injected with 200 ng Amp-specific dsRNA. Injection experiments were repeated three times for each dsRNA. Four to seven injected larvae were picked up from the sliced tuber at time interval of 12, 24, 48, 72 and 96 hours post injection for RNA isolation. Control larvae, injected with buffer, were treated as same as the experimental larvae. The extracted total RNA was quantified and cDNA was synthesized using Superscript II cDNA synthesis kit (Invitrogen) according to the manufacturer’s instruction.

qRT-PCR was carried out in a final volume of 20 µl reaction containing 10 µl SYBR^®^ Green I mix (Applied Biosystems, USA), 1 µl cDNA and 10 µM each gene specific forward and reverse primers (CHSRTF-361 and CHSRTR-489, Table 1). *Ph*. *operculella* actin gene was used as a reference control to normalize gene expression data where actin specific primer ACTF-135 and ACTR-419 were used (Table 1). qRT-PCR reactions were carried out in Mx3000 P Real-Time Thermocycler (Stratagene, La Jolla, CA, USA). The amplification conditions were: 10 min at 94 °C followed by 40 cycles of 15 sec at 95 °C and 1 min at 60 °C. The reaction was followed by dissociation curve analysis by heating at 95 °C for 60 sec; 55 °C for 30 sec and 0.2 °C increase per cycle till 95 °C. Three technical replicates were performed for each biological replicate. The relative gene expression data were calculated using the 2^−∆∆Ct^ method^36^. For each dsRNA fragment as well as Amp-dsRNA, fold change of *PhoCHSA* mRNA was calculated relative to the control at the same time interval. ANOVA test was performed in SPSS (20.0; IBM) to determine transcript level variation throughout time intervals for each dsRNA molecule. The P values < 0.05 were considered significantly different according to Duncan post hoc test and the results are presented as means ± SE.

## Results

### *PhoCHSA* sequence analysis

**Figure 1:**
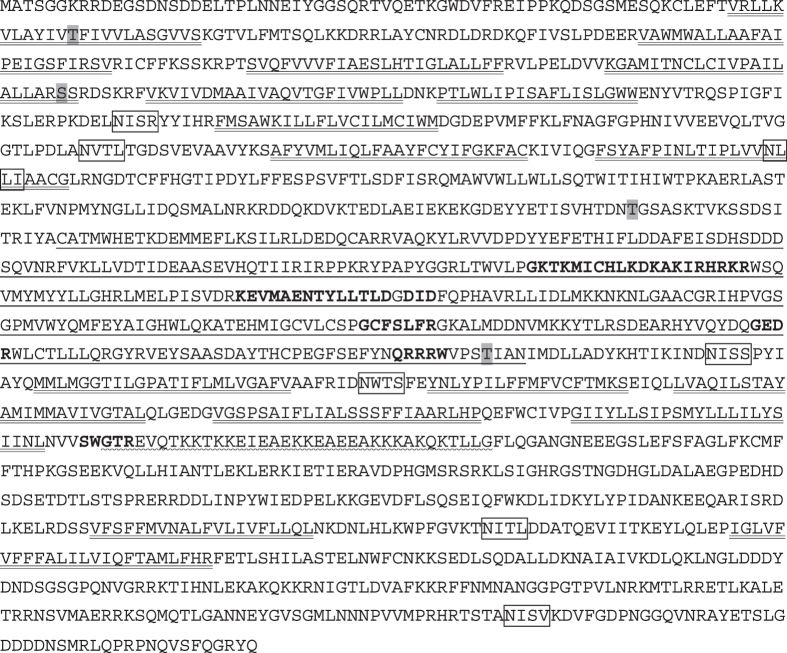
Deduced amino acid sequence of *Phthorimaea operculella* chitin synthase A. Transmembrane helices and spans are double underlined. Catalytic domain is single underlined. Coiled-coil domain is zigzag underlined. Conserved motifs necessary for enzyme function are shown in bold. N-glycosylation sites are enclosed in boxes and O-β-glycosylation sites are shaded in grey color.

The entire cDNA sequence of *PhoCHSA* (GenBank Acc. No. KU720384) was revealed by sequencing DNA fragments obtained by PCR reactions using cDNA prepared from epidermal tissues as the template. Initial RT-PCR using three degenerate primer sets produced three cDNA fragments of 110, 543 and 231 bp. The identity of the three fragments was verified by BLASTX and BLASTN on the GenBank database. Two intermediate regions of 2492 bp and 1205 bp were obtained by RT-PCR using specific primers designed based on the initial obtained sequences. The 5′ and 3′ regions were cloned and sequenced using 5′ and 3′ RACE, respectively. A total of 7 fragments (3 initial, 2 intermediate and 2 RACE fragments) were compiled together to produce the full-length cDNA of *PhoCHSA*. The full-length cDNA of *PhoCHSA* is 5,627 bp with 4,689 bp open reading frame coding for 1,563 amino acids (Fig. 1). The 5′ untranslated region (UTR) is 179 bp while the 3′ UTR is 759 bp including the TGA stop codon.

**Figure 2:**
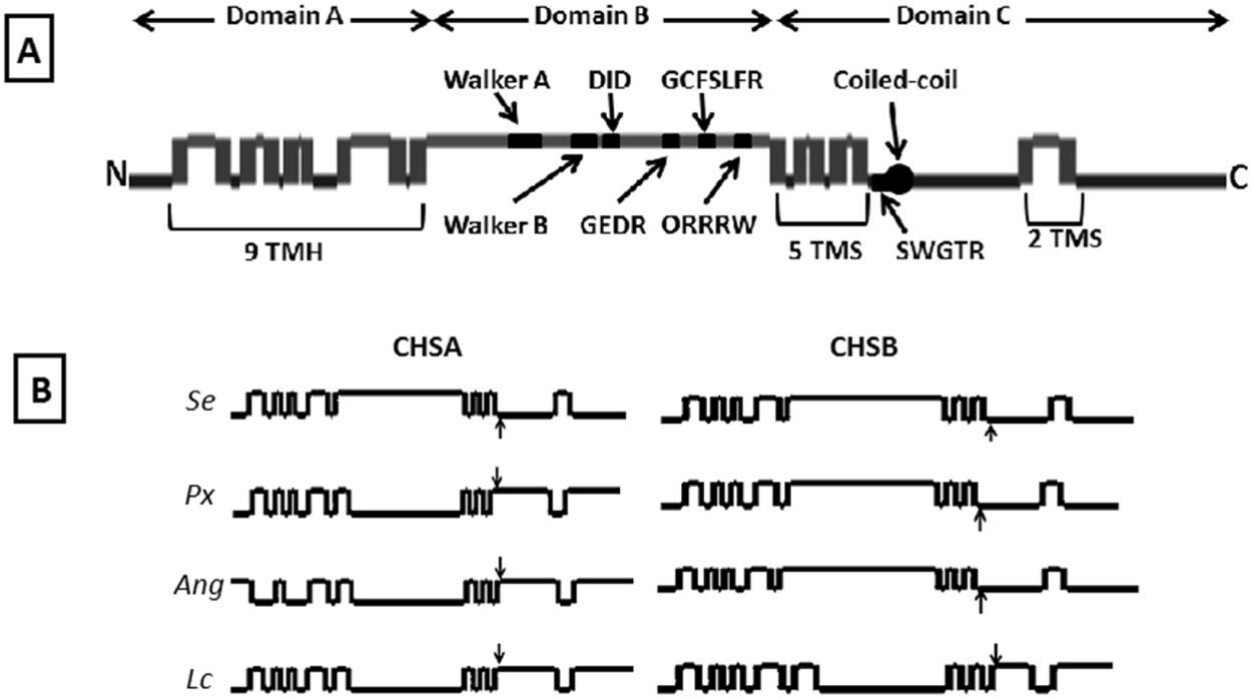
Predicted structure of PhoCHSA and selected CHS enzymes identified from other insects using TMHMM-2.0. (**A**) Topology of PhoCHSA showing the three major structural domains and associated structures and motifs. TMH: transmembrane helix, TMS: transmembrane span, N: N-terminus and C: C-terminus. Vertical bars represent the transmembrane regions while horizontal lines represent inner (lower) and outer (upper) domains. (**B**) Topology of selected CHS proteins showing the location of the catalytic domain and the SWGTR motif (indicated by arrow). Catalytic domains of *SeCHSA* (AAZ03545)*, SeCHSB* (ABI96087)*, PxCHSB* (XP_011565203) and *AngCHSB* (AGAP001205-PA) are located outside the plasma membrane while catalytic domains of *PxCHSA* (BAF47974)*, AngCHSA* (AGAP001748)*, LcCHSA* (AAG09712) and *LcCHSB* (KNC29062) are located inside the plasma membrane and facing the cytoplasm. Catalytic domain and SWGTR motif are always on opposite sides of the plasma membrane. *Ang*: *Anopheles gambiae*, *Lc*: *Lucilia cuprina*, *Px*: *Plutella xylostella*, *Se*: *Spodoptera exigua*.

**Figure 3:**
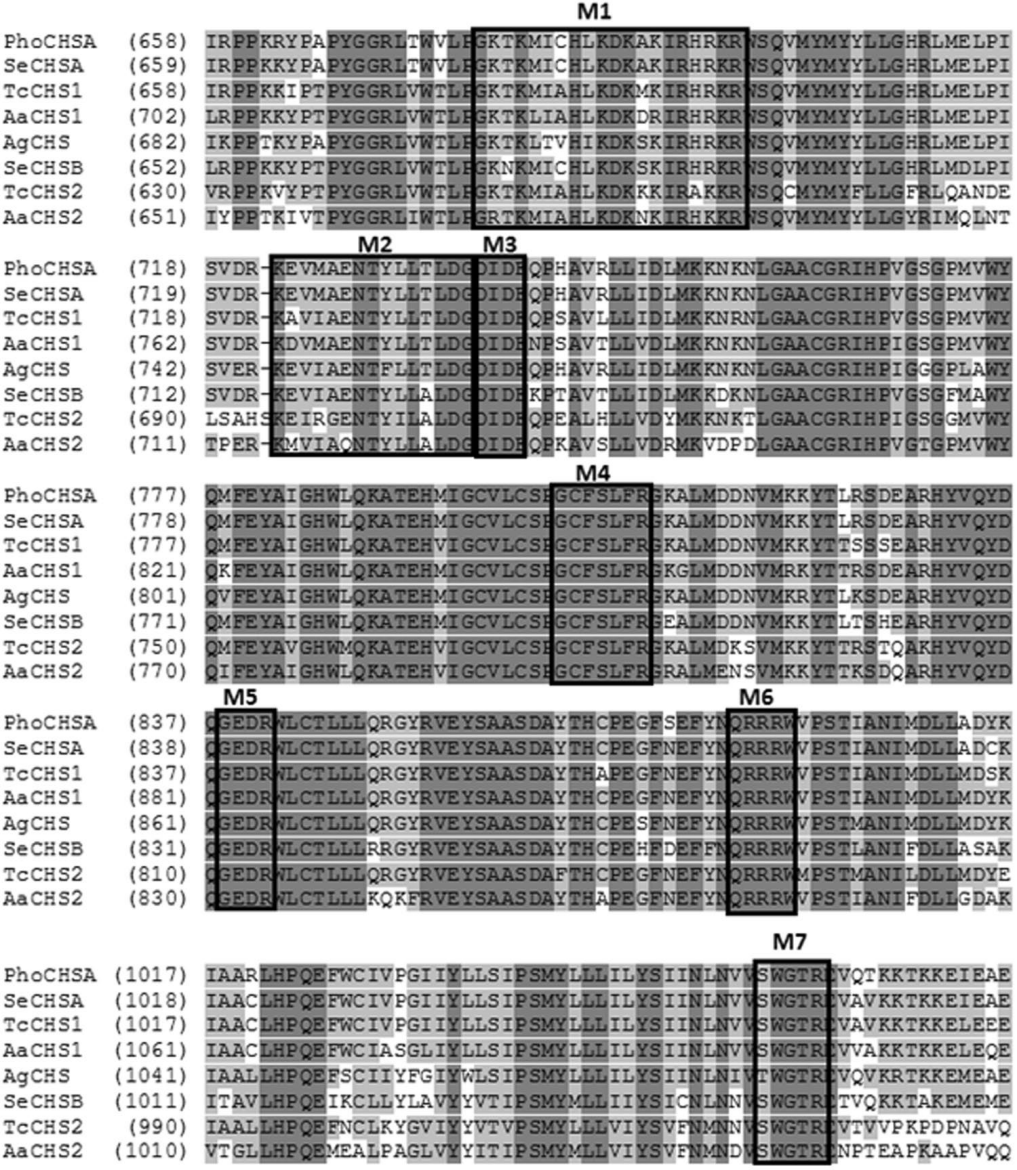
Partial amino acid alignment of PhoCHSA with selected CHS enzymes from other insects showing the conserved motifs found in all known insect CHS proteins. Identical residues are shown in dark grey background while conserved residues are shown in light grey background. M1 and M2: nucleotide binding sites similar to Walker A and Walker B motifs, M3 and M4: donor saccharide binding sites. M5: Acceptor binding site, M6: product binding site, M7: involved in chitin extrusion along with the 5TMS region (not shown). *Aa*: *Aedes aegypti*, *Ag*: *Aphis glycines*, *Se*: *Spodoptera exigua*, *Tc*: *Tribolium castaneum*, AaCHS1: AAEL002718-PA, AaCHS2: AAEL005618-PA, AgCHS: AFJ00066, SeCHSA: AAZ03545, SeCHSB: ABI96087, TcCHS1: NP_001034491, TcCHS2: NP_001034492.

No signal peptide was detected in the PhoCHSA predicted amino acid sequence and the predicted molecular weight of encoded protein is 178.3 kDa with isoelectric point (pI) of 6.5. Three conserved regions were detected in the predicted protein; N-terminus, middle and C-terminus domain (Fig. 2A). The N-terminus domain contains nine transmembrane helices detected by the TMHMM software. The middle domain is believed to be the catalytic domain and contains the following conserved sequences found in the GT2 family of glycosyltransferases to which chitin synthases belong: the UDP moiety binding sites GKTKMICHLKDKAKIRHRKR (similar to Walker A motif) and KEVMAENTYLLTLD (similar to Walker B motif), the donor saccharide binding sites DID (similar to DXD motif) and GCFSLFR (similar to GX_4_Y/FR motif), the acceptor saccharide binding site GEDR (similar to GEDRxxT/S motif), and the product binding site QRRRW (similar to Q/RXXRW motif) (Figs 1 and 2A). The C-terminus domain contains 7 transmembrane spans (TMS); 5 directly following the catalytic region and 2 closer to the C-terminus end of the protein (Fig. 2A). SWGTR conserved sequence was also detected in the C-terminus region after the 5^th^ TMS and at the beginning of a coiled-coil domain (in the region between 1060–1094 aa residues) predicted using the Pairciol program (Berger *et al*. 1995). The SWGTR domain along with the 5 TMSs are believed to play a role in chitin extrusion to outside the cell. Partial amino acid alignment of the catalytic region and the SWGTR motif surrounding region with similar regions from selected CHS proteins from different insect orders showed conserved domains reservation between PhoCHSA and both CHSA and CHSB proteins (Fig. 3).

A total of 13 glycosylation sites were predicted within the PhoCHSA protein (Fig. 1). Analysis using NetNGLyc 1.0 software revealed seven potential N-glycosylation sites at positions 291 (NISR), 358 (NVTL), 411 (NLTI), 904 (NISS), 943 (NWTS), 1304 (NITL) and 1517 (NISV). On the other hand, the YinOYang program predicted six O-β-glycosylation sites at positions 77 (T), 216 (S), 547 (T), 884 (T), 1370 (S) and 1557 (S).

**Figure 4:**
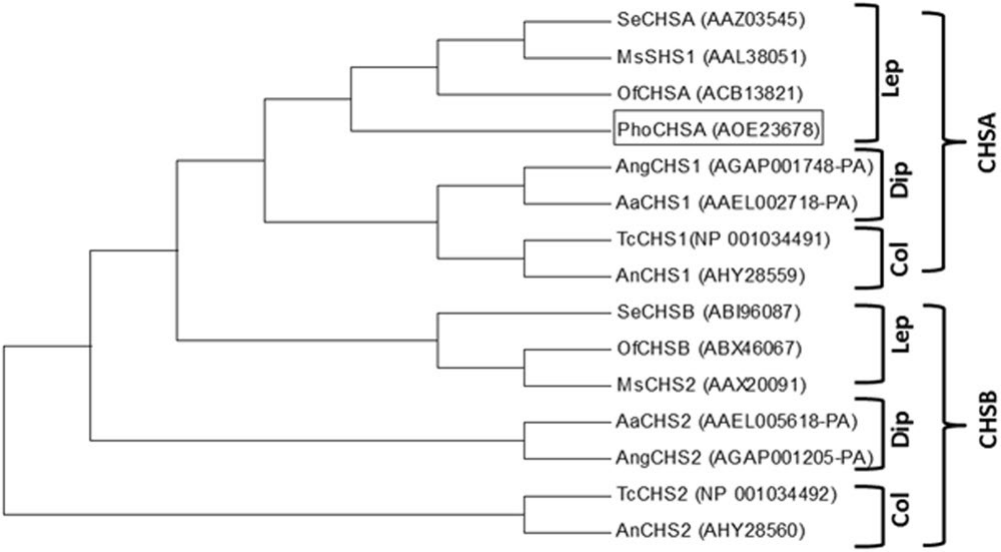
Phylogenetic analysis of PhoCHSA protein with CHSA (CHS1) and CHSB (CHS2) from selected lepidopteran, dipteran and coleopteran insects. PhoCHSA clustered with other CHSA proteins from insects and closely related to lepidopteran CHSA. Tree was generated by MEGA 7.0 using neighbor-joining method based on amino acid sequences. *Aa*: *Aedes aegypti*, *An*: *Anthonomus grandis*, *Ang*: *Anopheles gambiae*, *Ms*: *Manduca sexta*, *Of*: *Ostrinia furnacalis*, *Se*: *Spodoptera exigua*, *Tc*: *Tribolium castaneum*, Col: Coleoptera, Dip: Diptera, Lep: Lepidoptera.

Amino acid sequence comparison with other lepidopteran insects CHSA amino acid sequences indicated that PhoCHSA protein shares over 90% identities with *Ostrinia furnacalis* (ACB13821), *Spodoptera exigua*, (AAZ03545), *Helicoverpa zea* (ADX66429), *H. armigera* (AKJ54482), *Mamestra brassicae* (ABX56676) and *Cnaphalocrocis medinalis* (AJG44538). Also, it has 85–89% identities with *B. mori* (XP012549892), *Choristoneura fumiferana* (ACD84882), *Manduca sexta* (AAL38051), *Papilio Xuthus* (KPI93758) and *Ectropis obliqua* (ACA50098). Phylogenetic analyses showed that CHSA and CHSB from different insect orders are located at different phylogenetic groups. PhoCHSA is clustered with CHSA proteins from other insects and closely related to lepidopterans which confirm its identity as a member of class A chitin synthases (Fig. 4).

### dsRNA bioassay

**Figure 5:**
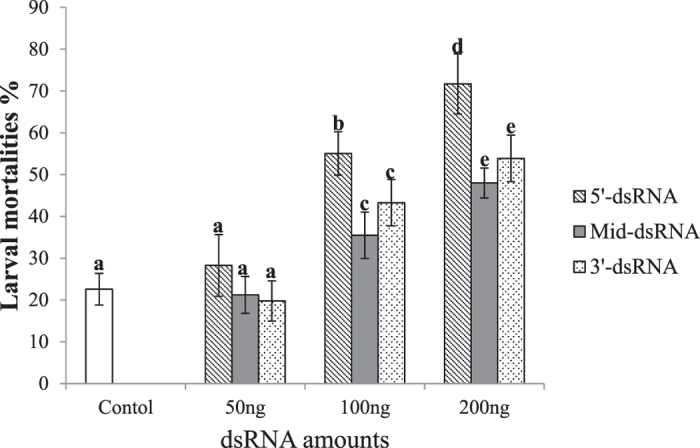
Larval mortality after injection with three amounts (50, 100 and 200 ng) of three different dsRNA molecules; 5′-dsRNA, Mid-dsRNA and 3′-dsRNA targeting PhoCHSA mRNA. dsRNA was injected into 3^rd^ larval instars of potato tuber moth and larval mortality was recorded 120 hours post-injection. Error bars represent ±SE. Within the same dsRNA concentration different letters indicate significantly different values at P < 0.05.

**Figure 6:**
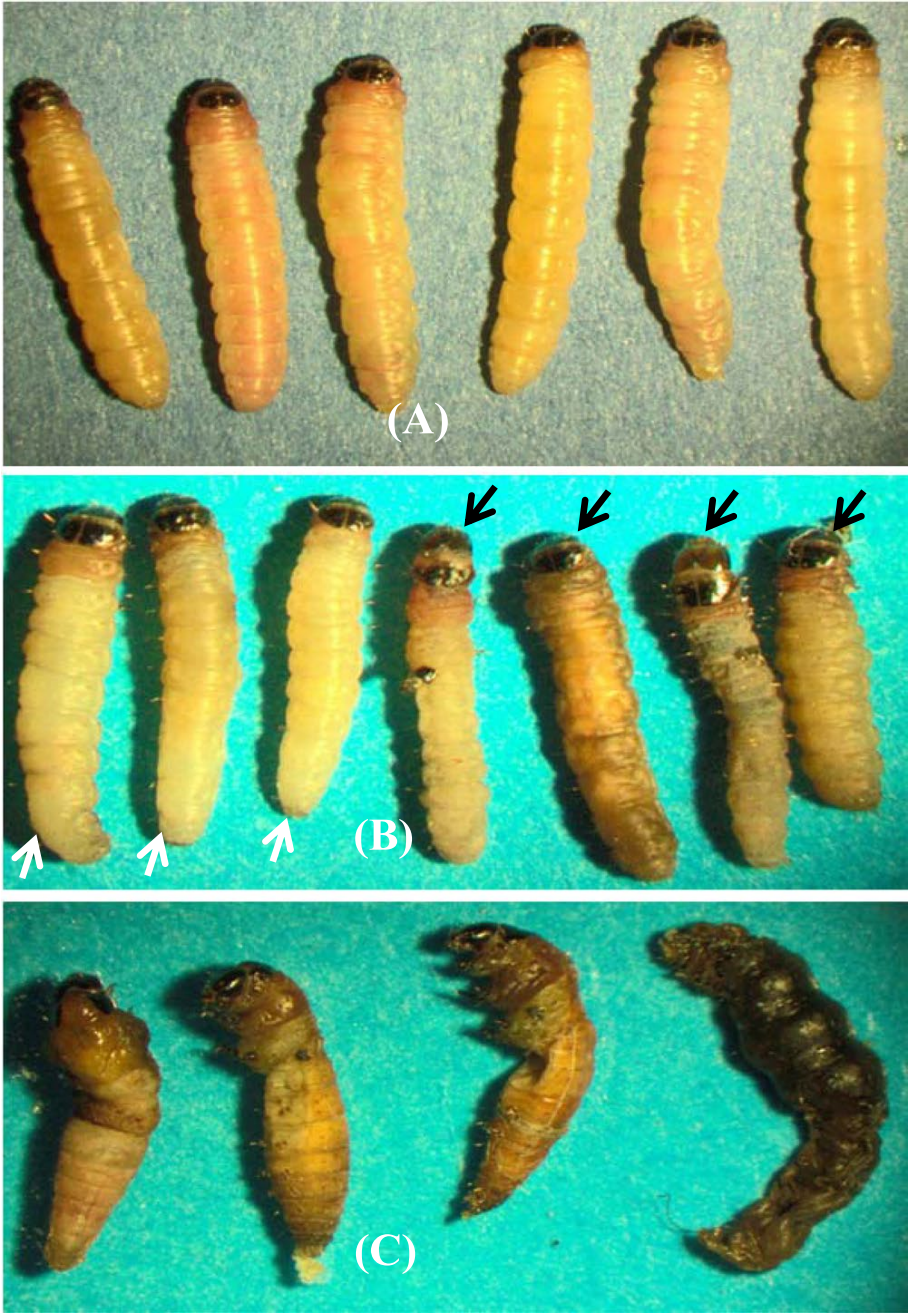
Effects of PhoCHSA RNA interference. Two-day-old 3rd instar larvae were injected with 200 ng of Mid dsRNA, 3′-dsRNA or 5′-dsRNA targeting PhoCHSA mRNA. Larval mortalities and abnormalities were detected at 120 hours post-injection. The figures represent larvae treated with 5′-dsRNA. Control larvae injected with dsRNA-free buffer show normal development (**A**). Some of the injected larvae are either died (black arrows) or show slower development of larvae (white arrows) (**B**) and others failed to pupate (**C**).

To determine the effect of silencing *PhoCHSA* on PTM, larvae were injected with three different amounts of three dsRNA targeting different regions of the transcript. Mortality within the first 24 hours was neglected to avoid handling damage and injection injury that occurred during the injection procedure. Larval mortality was recorded after five days from initial injection time (Fig. 5). Mortalities caused by 50 ng of each dsRNA were similar to the controls. 100 ng of dsRNA resulted in mortalities of 55% ± 5.2 for 5′-dsRNA, 35.4% ± 5.5 for Mid-dsRNA and 42.8% ± 5.9 for 3′-dsRNA. Mortalities were different from control and 5′-dsRNA gave the highest mortality which was different from the other two groups. The percentages of larval mortalities due to injection with 200 ng of 5′-dsRNA, Mid-dsRNA and 3′-dsRNA were 71.7% ± 7.2, 47.8% ± 3.5 and 53.8% ± 6.8, respectively (Fig. 5). dsAmpR caused mortality of 22.8% ± 3.7. The high mortality within control is more likely due to failure of larvae to re-penetrate the tuber slices; an observation from our experience with the potato tuber moth. However, statistical analysis showed that mortality caused by target gene dsRNA was significantly different from the dsAmpR mortality (P < 0.05) and 5′-dsRNA mortality was significantly different compared to the other two dsRNAs (Mid-dsRNA and 3′-dsRNA). Mortality caused by dsRNA depended on the delivered dose where increasing the dose caused higher mortality for the three dsRNA constructs. Furthermore, some of the treated larvae were morphologically deformed compared to the control (Fig. 6). The whole insect body was trapped by old cuticle and eventually died. Shrinkage of the bodies and slower development were also displayed of treated larvae (Fig. 6B). Few larvae were unable to pupate (Fig. 6C) and cannot dispose the old cuticle.

### Quantitative real time PCR Analysis

#### Expression pattern of *PhoCHSA*

**Figure 7:**
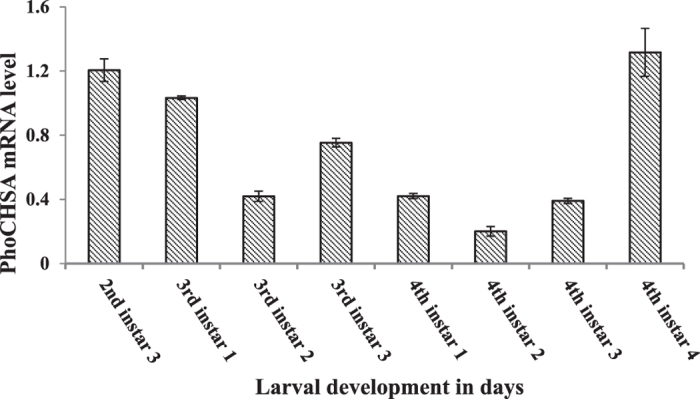
Expression profile of chitin synthase A in *Ph. operculella* during larval development between the last day of 2nd and 4th instars. *PhoCHSA* mRNA was measured by qRT-PCR during the developmental stages of larvae. The actin gene was used as reference gene in qRT-PCR. Error bars represent ±SE.

High levels of *PhoCHSA* mRNA expression were detected in the last day of the 2nd, 3rd and 4th stadium (Fig. 7). The expression levels declined gradually at day 1 of the following instar and continue to the minimum followed by dramatic increase. PhoCHSA expression is observed during periods of larval-larval and larval-pupal phases, meanwhile, the insect requires large amount of chitin to synthesize new cuticle.

#### RNAi

**Figure 8:**
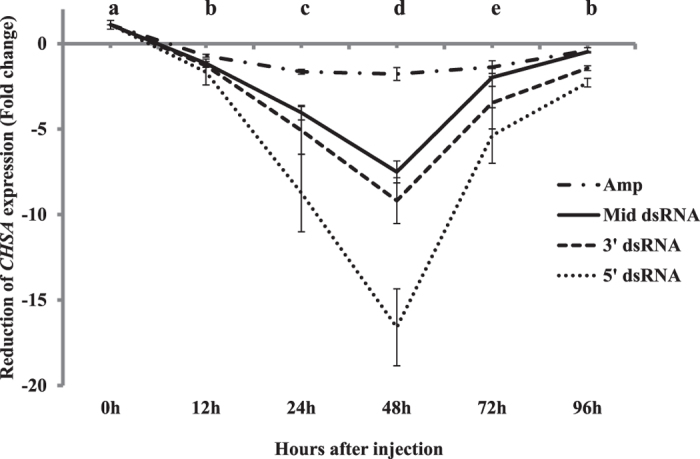
Analysis of c*hitin synthase A* silencing through RNAi in *Ph. operculella* using qRT-PCR. The efficiency of three dsRNA molecules targeting different sites on *PhoCHSA* gene; 5′-dsRNA, Mid-dsRNA and 3′-dsRNA, is expressed as fold difference of gene expression relative to control. The expression of actin gene was used as an endogenous reference gene to normalize the data and to calculate the fold change using 2^−ΔΔct^ method. All values are mean ± SE, n = 3 (n; number of technical replicates). Different letters indicate significantly different values at P < 0.05.

Time course effect of dsRNA on the level of *CHSA* mRNA was analyzed by quantitative real-time PCR using *actin* mRNA to normalize the data. The qPCR analysis indicated that all three fragments caused reduction of the *CHSA* mRNA level with different values (Fig. 8). The following 12 h after injection showed the lowest effect of dsRNA on *CHSA* transcript level throughout the experiment. Within the first 24 h post-injection, there was a reduction among the tested dsRNAs with a range of four to eight folds. 5′-dsRNA generated the highest suppression effect compared with Mid-dsRNA and 3′-dsRNA (P < 0.05; Duncan’s test; n = 3, where n is number of technical replicates). At 48 h, 5′-dsRNA caused 16.59 ± 2.5 fold reduction of *CHSA* mRNA level while only 7.5 ± 0.64 and 9.2 ± 1.34 reduction fold were detected for Mid-dsRNA and 3′-dsRNA, respectively. *PhoCHSA* expression gradually restored its normal level at 96 hours. The estimated *CHSA* mRNA of the survived larvae at 96 h post-injection of 5′-dsRNA, Mid-dsRNA and 3′-dsRNA was lower than control by only 2.3 ± 0.25, 0.47 ± 0.08 and 1.4 ± 0.13 fold, respectively. The lowest *CHSA* mRNA level detected in larvae injected with Amp-specific dsRNA was 1.77 ± 0.37 fold at time 48 hours. These results suggest that the knockdown effect of the dsRNA reached its peak within 2 days from initial dose and its influence reduced dramatically within the following days. Moreover, selecting the target site on the desired genes for dsRNA plays an important role in RNAi effectiveness.

## Discussion

Chitin biosynthesis in insects starts with trehalose sugar, mainly found in the insect hemolymph, and ends with the chitin polymer. Chitin synthases are highly conserved membrane bound enzymes in organisms that synthesize chitin where they utilize the activated sugar “UDP-*N*-acetylglucosamine” to form the chitin polymer^37^. Two forms of chitin synthase are present in most insect species; chitin synthase A and chitin synthase B. Chitin synthase A is expressed mainly in the epidermal cells where it is responsible for the synthesis of chitin incorporated in the cuticle. Chitin synthase B is expressed in midgut epithelial cells as it is responsible for the synthesis of chitin incorporated in the PM^4, 38^. In this study, full-length cDNA of chitin synthase A was cloned from larvae of the potato tuber moth. The full-length cDNA of PhoCHSA is 5,627 bp with 4,689 bp open reading frame coding for 1,563 amino acids which is in the range of class A chitin synthase genes isolated from other insects. Structural analysis of the deduced amino acid sequences indicated the presence of three distinct domains; N-terminus, middle and C-terminus, a feature of all known CHS enzymes isolated from insects. The N-terminus domain is hydrophilic containing nine transmembrane helices. The N-terminal region is less conserved among insects but the transmembrane helices seem to be necessary for the enzyme activity^39^. The central region is believed to contain the catalytic domain which contains highly conserved motifs that characterize chitin synthases identified from different phyla such as the walker A and walker B like domains, two donor saccharide binding sites, the acceptor saccharide binding site, and the product binding motif^6, 7^. Zhu *et al*.^40^ suggested that the catalytic domain is facing the cytoplasmic side of the plasma membrane where the substrate is found. Topological analysis showed that the catalytic domain of PhoCHSA is located on the outer side of the plasma membrane and not facing the cytoplasm, opposite to what was suggested by Zhu *et al*.^40^. The C-terminal region contains 5 TMS located directly after the catalytic region with two more TMS located closer to the C-terminus of the protein, a conserved arrangement found in all known CHS enzymes isolated from insects. The SWGTR domain along with the 5 TMS are believed to play a role in chitin extrusion to outside the cell similar to the proposed mechanism of cellulose extrusion^41^. This suggested role of the C-terminus TMS region needs to be reviewed especially when the catalytic region is predicted to be located to the outside of the plasma membrane. We noticed that the catalytic domain and the SWGTR motif are always located on different membrane sides (Fig. 2), the significance of such arrangement is not known.

RNAi is a molecular biology technique that is used to study gene function, also it has been adopted as emerging pest control technology. Early attempts to use RNAi for insect control were reported by Mao *et al*.^27^ and Baum *et al*.^17^. They produced transgenic plants expressing dsRNA against insect genes that showed protection against two major pests from two different insect orders; the cotton bollworm *Helicoverpa armigera* (Lepidoptera) and the western corn rootworm *Diabrotica virgifera virgifera* (Coleoptera). These two reports opened the door for many trials targeting different insect genes by different methods such as injection, soaking, feeding, transgenic plants and others; such trials were summarized in some reviews such as Kola *et al*.^22^ and Kim *et al*.^42^. Also the challenges, the future and recommendations for successful insect control by RNAi were discussed in some reviews such as Scott *et al*.^21^ and Burand and Hunter^43^. Chitin synthesis is an attractive target of some insecticides because the chitin synthesis pathway does not exist in vertebrates and plants^14^. However, resistance and non-target toxicity affected their use in insect and mite control^14, 44^. For replacing those chemicals with safer approach, targeting chitin synthase genes by RNAi showed some success in initial studies aiming for insect control. In insects containing two CHS genes, targeting both genes was lethal to the insects in different life stages and affected embryos and egg laying. For example, RNAi for *TcCHS1* in the immature stages of the red flour beetle *Triboliun castaneum* disrupted molting while RNAi of *TcCHS2* caused the larvae to stop feeding and to have small body size^4^. In another report on the same insect injection of dsRNA of *TcCHS1* into immature adults was lethal to both males and females while injection in mature females caused abnormalities in embryos that couldn’t hatch from eggs^45^. In another coleopteran, the cotton boll weevil *Anthonomus grandis*, injection of *CHS1* dsRNA in adult female caused head capsule and mandible malformation in embryos which couldn’t hatch from eggs and when mechanically hatched could not feed and died^46^. RNAi for CHS genes in lepidopteran pests was also conducted and showed some success. For example, feeding *Spodoptera exigua* larvae on artificial diet containing *E. coli* bacterial cells expressing *SeCHSA* dsRNA caused lower survival rates in 4^th^ and 5^th^ instar larvae, prepupae and pupae^47^. Jin *et al*.^31^ developed tobacco plants producing dsRNA for the *Helicoverpa armigera CHSA* in the plastids. When they fed *H. armigera* larvae on the transplastomic tobacco leaves producing *HaCHSA* dsRNA, larvae had lower body weight, reduced growth and pupation rates compared to controls. Hemipteran insects seem to have only one CHS gene belongs to class A^11, 31, 48^. In the case of the brown citrus aphid *Toxoptera citricida*, fourth instar nymphs fed on *TCiCHS* dsRNA showed lower molting rate, mortality during molting and lower CHS expression level compared to control larvae^11^. In the blood feeding Chagas disease vector, *Rhodnius prolixus*, injection of *RpCHS* dsRNA into 5^th^ instar nymphs led to abnormal ecdysis and the majority of the nymphs failed to molt to adults while injection into adult females led to reduction in oviposition and most of the embryos died inside the eggs and did not hatch^31^. In this report, we showed that injection of *PhoCHSA* dsRNA into third instar larvae of the potato tuber moth is lethal. This lethality is most likely due to the effect of RNAi which was confirmed by qRT-PCR analyses. In general, qRT-PCR analyses showed reduction in the level of CHS transcript compared to CHS transcript level in the control group. It is also clear that selection of the RNAi target region of the gene is critical to achieve the best silencing effect. We selected three regions of the CHS gene to target by RNAi and targeting the 5′ region of the gene produced the highest mortality compared to the other 2 regions (middle and 3′ region). Moreover, qRT-PCR analyses showed that targeting the 5′ region gave the highest reduction in CHS transcript level, in accordance with the highest mortality level noticed in the toxicity bioassay. Similar results were reported by Perkin *et al*.^49^ where they got different silencing rates of the cathapsin L gene by targeting different regions of the gene by RNAi. They found that targeting the 3′ region produced the most silencing effect and assumed that the 3′ region is the catalytic domain of the enzyme. In the current study, targeting the catalytic domain (middle) and the probable extrusion domain (3′) produced less silencing and, hence, less mortality than targeting the 5′ transmembrane helices domain although the later domain is not included in the enzymatic catalysis. The differences in the silencing effect are probably due to the structure of the mRMA itself. The secondary structure of the mRNA target site plays an important role in RNAi efficiency^50, 51^. Target sites on the mRNA with large number of unpaired nucleotides are highly accessible for siRNA. In contrast, target sites with high number of bound nucleotides are less accessible for siRNA and therefore reduce the knockdown efficiency. Yiu *et al*.^52^ found that target sites on or near to bigger loops or branches to be less effective for RNAi.

Our study also showed the effect of silencing on the CHS transcript level over 96 h post-treatment. The silencing effect started 24 h, peaked 48 h, and recovered 96 h post-injection. The peak effect of injected dsRNA is more likely dose and insect species-dependent. *T. castaneum* larvae injected with dsRNA of lethal giant larvae gene (TcLgl) showed significant suppression of the TcLg1 transcripts 2 days post-injection^53^. In the brown plant hopper, *Nilaparvata lugens*, knockdown of two trehalose-6-phosphate synthases caused inhibition of mRNA expression of twelve chitinase genes 48 h after treatments and up to 72 h for other genes^54^. Whereas, the maximum silencing of Cytochrome P450 derivative (CYP6) and Aminopeptidase N (APN) of the rice yellow stem borer was observed at 12^th^ and 15^th^ day of treatment, respectively^55^.

In summary, we isolated the CHSA cDNA from *Ph. operculella*. Translated PhoCHSA protein contains basic domains and features of CHS enzymes identified from other insects. Phylogenetic analysis confirmed that PhoCHSA belongs to the class A CHS enzymes. We also showed that PhoCHSA RNAi is lethal to PTM and results were confirmed by qRT-PCR. Selection of the RNAi target region is important where different regions gave different silencing effects. Our results suggest that PhoCHSA could be a suitable target gene for RNAi application in PTM control. In our effort to utilize these results, we are developing transgenic potato plants expressing CHS dsRNA and its testing is currently underway.
